# Supplementary material for: Evolution of a fuzzy ribonucleoprotein complex in viral assembly
Source: eLife. 2025 Dec 30;14:RP108922. doi: 10.7554/eLife.108922 (PMC12753105; doi:10.7554/eLife.108922)
Supplement: Figure 4—figure supplement 1—source data 1. [file elife-108922-fig4-figsupp1-data1.zip › Figure 4-figure supplement 1-source data 1/Figure 4-figure supplement 1-source data 1.pdf]

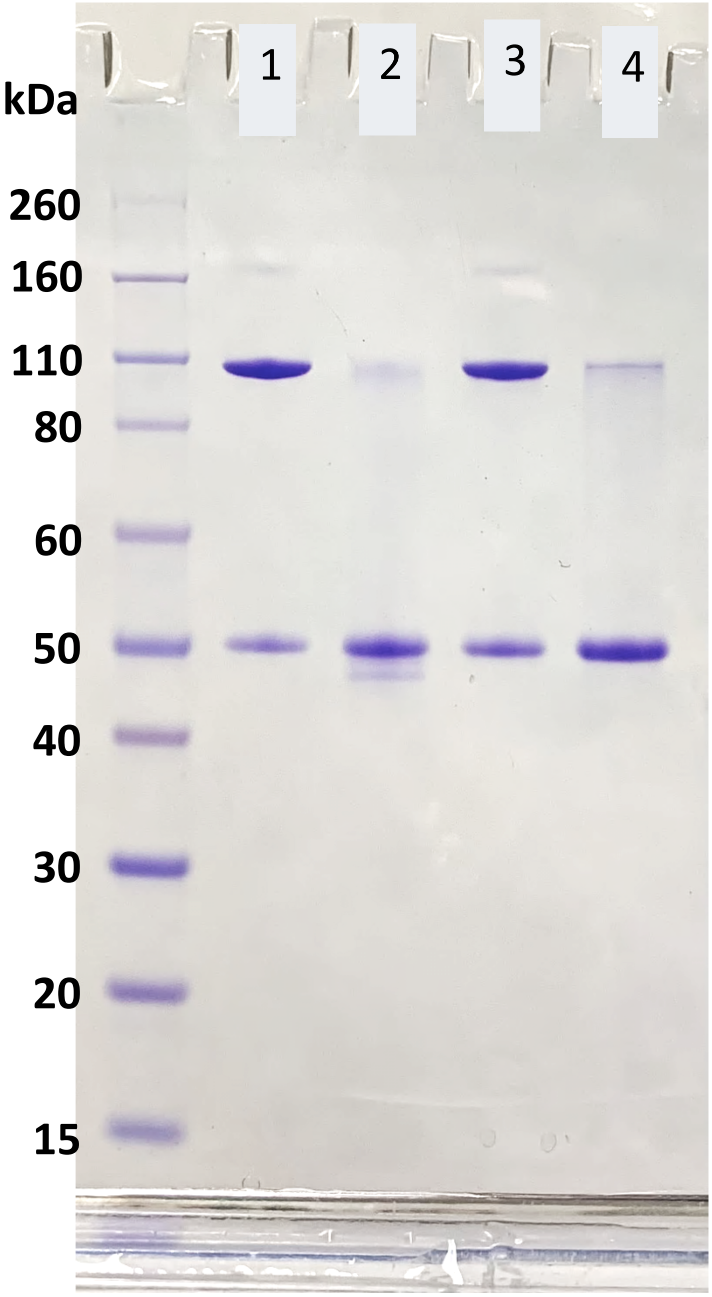

Figure 4-figure supplement 1-source data 1: Non-reducing SDS-PAGE of reduced and oxidized N:G215C* and N_λ*. Lanes: 1) oxidized N_λ*; 2) reduced N_λ; 3) oxidized N:G215C*; 4) reduced N:G215C.
