# Supplementary figures and images for: Evolution of a fuzzy ribonucleoprotein complex in viral assembly
Source: eLife. 2025 Dec 30;14:RP108922. doi: 10.7554/eLife.108922 (PMC12753105; doi:10.7554/eLife.108922)

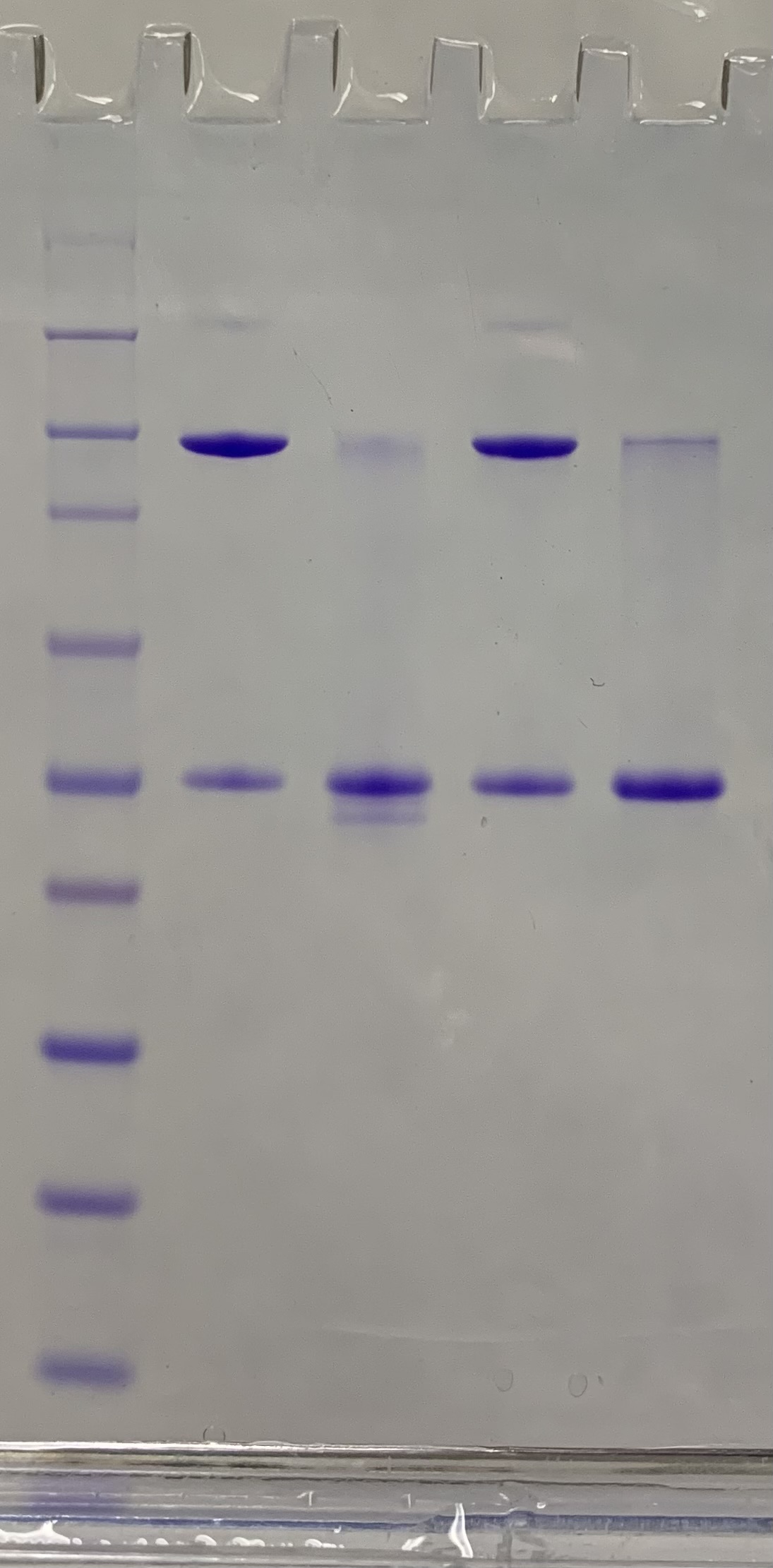

Supplement: Figure 4—figure supplement 1—source data 2. [file elife-108922-fig4-figsupp1-data2.zip › Figure 4-figure supplement 1-source data 2/Figure4-figure supplement 1-source data 2.jpg]
