# Supplementary material for: Evolution of a fuzzy ribonucleoprotein complex in viral assembly
Source: eLife. 2025 Dec 30;14:RP108922. doi: 10.7554/eLife.108922 (PMC12753105; doi:10.7554/eLife.108922)
Supplement: Supplementary file 1. [file elife-108922-supp1.docx]

**Table S1. Peptide and Oligonucleotide Sequences**

| designation | sequence |
| --- | --- |
| N_1-43_ (N-arm) | MSDNGPQNQRNAPRITFGGPSDSTGSNQNGERSGARSKQRRPQ |
| N_1-43_:P13L | MSDNGPQNQRNALRITFGGPSDSTGSNQNGERSGARSKQRRPQ |
| N_1-43_:P13L,Δ31-33 | MSDNGPQNQRNALRITFGGPSDSTGSNQNG---GARSKQRRPQ |
| N_210-246_ (LRS) | MAGNGGDAALALLLLDRLNQLESKMSGKGQQQQGQTV |
| N_210-246_:G214C | MAGNCGDAALALLLLDRLNQLESKMSGKGQQQQGQTV |
| N_210-246_:G215C | MAGNGCDAALALLLLDRLNQLESKMSGKGQQQQGQTV |
| T_10_ | TTTTTTTTTT |
| SL7 | ﻿acguggcuuuggagacuccguggaggaggucuuaucagaggcacgu |
